# Co-expression of intermediate filaments glial fibrillary acidic protein and cytokeratin in pituitary adenoma

**DOI:** 10.1007/s11102-020-01087-3

**Published:** 2020-10-01

**Authors:** Nina Wiesnagrotzki, Christian Bernreuther, Wolfgang Saeger, Jörg Flitsch, Markus Glatzel, Christian Hagel

**Affiliations:** 1 Institute of Neuropathology, University Medical Center Hamburg-Eppendorf, Martinistr. 52, 20246 Hamburg, Germany; 2 Institute of Pathology, University Medical Center Hamburg-Eppendorf, Martinistr. 52, 20246 Hamburg, Germany; 3 Department of Neurosurgery, University Medical Center Hamburg-Eppendorf, Martinistr. 52, 20246 Hamburg, Germany

**Keywords:** Pituitary adenoma, Glial fibrillary acidic protein, Cytokeratin, Folliculostellate cells

## Abstract

**Purpose:**

To analyze the co-expression of the intermediate filaments GFAP and cytokeratin in 326 pituitary adenomas with regard to the distribution pattern, the subtype of the adenoma and clinical prognostic data.

**Methods:**

Tissue from 326 pituitary adenomas and 13 normal anterior pituitaries collected in the Institute of Neuropathology, University Medical Center Hamburg-Eppendorf, between 2006 and 2009 was investigated by immunohistochemistry, immunofluorescence and electron microscopy.

**Results:**

Co-expression of intermediate filaments GFAP and cytokeratin was associated with hormone expression in 62/278 cases (22%), but only found in 2/48 (4%) of null cell adenomas (p < 0.01). Simultaneous co-expression of GFAP and cytokeratin in the same cells was demonstrated in 26 out of 326 pituitary adenomas and in all 13 pituitaries. In pituitary intermediate filaments were demonstrated in a larger area of the cytoplasm than in adenoma (p < 0.01), however, overlapping expression was seen in 2.6% of the total area in both, pituitary and adenoma. Congenially, cells with overlapping expression were found near vessels and in follicles. Furthermore, adenomas with cellular co-expression of GFAP and cytokeratin were associated with a lower recurrence rate (7.7%) compared to adenomas without co-expression of intermediate filaments (17.8%).

**Conclusions:**

Cellular co-expression of the intermediate filaments GFAP and cytokeratin in pituitary adenomas and the pituitary was demonstrated and shown to be associated with hormone expression and low recurrence rate. The results are discussed with regard to the biology of folliculostellate cells, neural transformation and tumor stem cells. This study may complement the understanding of pituitary adenoma biology.

**Electronic supplementary material:**

The online version of this article (10.1007/s11102-020-01087-3) contains supplementary material, which is available to authorized users.

## Introduction

The pituitary adenoma represents the most frequent space occupying lesion in the sella turcica and accounts for 10–15% of all intracranial neoplasias. Most often it is of monoclonal origin and appears monohormonal but may also be bi- or plurihormonal or hormone inactive. The cell of origin determines the clinical and biochemical phenotype of the adenoma. Tumours deriving from lactotroph (PRL), somatotroph (STH), thyreotroph (TSH) (transcription factor PIT1), corticotroph (ACTH) (transcription factor TPIT), or gonadotroph (LH, FSH) (transcription factor SF1) cells are therefore characterized by an autonomous, pathologically increased hormone secretion with reduced response to physiological inhibition. Plurihormonal adenomas are derived either from a single polysecretory cell or from different cells within the same tumour.

Intermediate filaments are attributed to a variety of functions, mainly cell plasticity, motility and stability. These include the intracellular organization of organelles (“compartmentalization”), signal transduction, cell polarity, cell plasticity, gene regulation or stress absorption in the case of toxic damage. Cyto-architecture depends on cell activity. Time-lapse images show that the network of intermediate filaments is highly dynamic. Despite their tissue specificity, occasional co-expression of different intermediate filaments is observed [1]. *Ogawa *et al*.* [2] described the co-expression of keratin and neurofilament (NF) in endocrine cells of healthy anterior pituitary and co-expression of keratin, vimentin and GFAP in the epithelial cells of the pars intermedia, where the cells form cystic follicles.

In 1953, *Rinehart and Farquhar* [3] first described stellate cells in the adenohypophysis, later also named folliculostellate cells [4]. With their multiple cytoplasmic processes, the folliculostellate cells form networks with each other and with the epithelial cells via desmosomes or gap junctions [5]. Yet, both, the origin and function of folliculostellate cells are not fully understood. *Velasco *et al*.* [6] and *Morris and Hitchcock* [7] compared the folliculostellate cells to glial cells because of their similar morphology and antigen expression pattern which includes S-100, GFAP, and vimentin [8–10]. They are attributed control of paracrine functions in hormone synthesis and secretion as well as physiological and neoplastic cell proliferation [5, 10, 11]. Folliculostellate cells produce a variety of factors that alter the functions of neighbouring cells and express receptors for pituitary hormones.

This present retrospective study was undertaken to investigate the expression of intermediate filaments GFAP and cytokeratin in pituitary adenoma with relation to hormone expression and clinical parameters.

## Materials and methods

### Patient data and tissue specimens

The cohort comprised 326 patients (171 female, mean age 47.2 years; 154 male, mean age 51.0 years; 1 missing value). In 52 cases a recurrence occurred (see Table 1 in Supplement).

The tumour samples were routinely assessed for proliferation index by Ki-67-immunohistochemistry and for expression of hormones (STH, PRL, ACTH, FSH, LH, TSH, see Table 1 in Supplement). Besides the 326 samples of pituitary adenoma, 13 specimens that contained regular pituitary only were included. Among the adenomas, there were 62 samples with co-expression of intermediate filaments GFAP and cytokeratin. By light microscopy 26 of these samples presented with supposedly overlapping expression of GFAP and cytokeratin (see Table 2 in Supplement). The remaining 36 tissue samples showed no signs of co-localization of intermediate filaments. The paraffin blocks of the selected 26 pituitary adenomas, as well as the 13 pituitary specimens, were further analysed by additional H&E stains and single as well as double labelling immunofluorescent staining with antibodies against GFAP and broad spectrum cytokeratin.

### Immunofluorescence

Paraffin sections of 4 µm thickness were cut, pre-treated by heating in a micro wave oven for 20 min in citrate buffer pH 6 followed by incubation with primary antibodies in blocking solution over night at 4 °C (GFAP: DAKO #Z 0334, polyclonal, rabbit, dilution 1:500; cytokeratin: broad-spectrum clone KL1 Immunotech #1918, mouse, dilution 1:200). On the following day fluorescent secondary antibodies were applied (GFAP donkey anti-rabbit IgG Al 594 (red): Molecular Probes #A32754, dilution 1:200; cytokeratin donkey anti-mouse IgG AI 488 (green): Molecular Probes #A21206, dilution 1:100) according to standard protocols. In the consecutive step, some samples were stained with DAPI (DAPI-Fluoromount-G, Southern Biotech #0100-20).

**Fig. 1:**
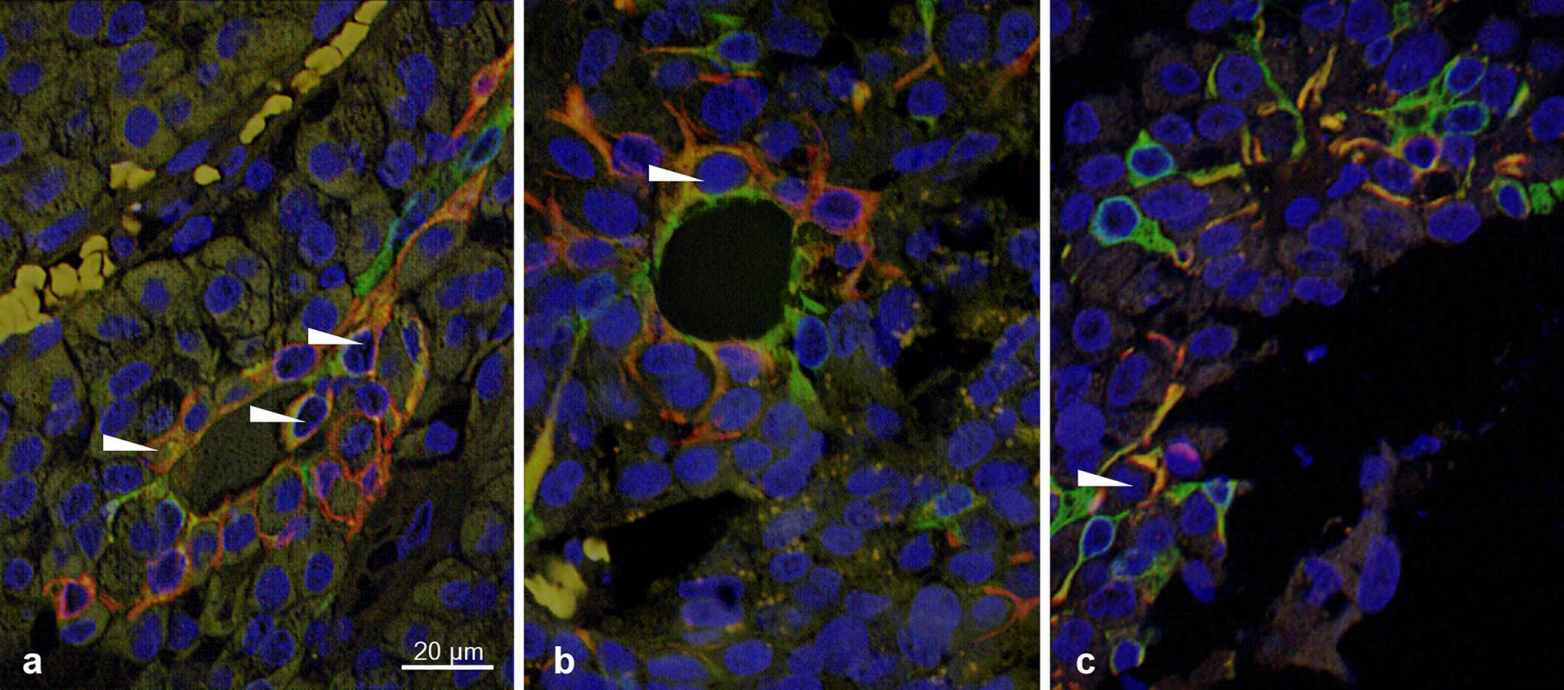
Immunofluorescence microscopy of adenoma with GFAP and cytokeratin co-expressing cells (arrows): **a** next to vessel; **b** in follicular formation; **c** diffusely distributed

For quantitative assessment, the specimens were evaluated with a fluorescence microscope (Zeiss Axiovert 200 M, objective Plan Neofluar 63 x/1.25 oil, Reflector: 43 HE Ds Red, camera: Apotome Cam). Photomicrographs of green and red areas in each sample as well as of overlapping areas appearing in yellow were measured in pixel^2^ in Photoshop version 7. Areas were manually selected using the magic wand tool and areas were related to each other (green/yellow, red/yellow; Fig. 1).

### Electron microscopy

For electron microscopic analyses, paraffin-embedded adenoma tissue was de-waxed and fixed in glutaraldehyde followed by incubation in chrome-osmium according to Dalton for 2 h, dehydrated in ethanol and embedded in Epon 812 (Serva). After polymerization of the resin semithin sections of 1 µm were cut and stained with toluidine blue. Appropriate specimens were further processed for electron microscopy by cutting ultrathin sections of 80 nm thickness that were counterstained with uranyl acetate (Polyscience, Eppelheim, Germany) and lead citrate (Riedel-de Haën, Seelze, Germany). The preparations were analyzed with a LEO 912 AB OMEGA electron microscope (Leo Elektronenmikroskopie, Oberkochen, Germany).

### Statistics

All statistics were performed as exploratory analyses. Statistical computations were performed using SPSS for Windows version 25. Methods comprised two-sided correlation analysis according to Pearson for metric variables and correlation analysis according to Kendall for non-metric data. Further Mann-Whitney-U-test and chi-square were used for nominal scaled variables. P values < 0.05 two-sided were regarded as significant.

## Results

### Intermediate filament expression

Evaluation of the 326 pituitary adenomas revealed 62 cases (19%) with co-expression of GFAP and cytokeratin. By light microscopy, 26 samples of this subset presented a likely co-localization of GFAP and cytokeratin. In the control group, all 13 samples of pituitary anterior lobe tissue expressed GFAP and cytokeratin (see Table 2 in Supplement).

### Intermediate filament expression, proliferation and adenoma recurrence

Ki-67-proliferation index (mean 1.64%) neither correlated with tumour recurrence or intermediate filament expression (see Table 3 in Supplement). Also detection of GFAP and/or cytokeratin was not associated with adenoma relapse. However, 62 tumours that showed a co-expression of GFAP and cytokeratin reoccurred significantly less frequently than those that only expressed one or no intermediate filament (5/62 vs. 47/264, see Table 1 in Supplement).

### Intermediate filament expression and hormone expression

Cellular co-expression of intermediate filaments GFAP and cytokeratin was associated with hormone expression in all 26 cases, and was not found in any of null cell adenomas. Further analysis revealed that cytokeratin expression, GFAP expression and co-expression of cytokeratin and GFAP correlated significantly with STH and PRL detection.

### Intermediate filament expression in normal anterior pituitary and adenoma

The area with overlapping expression of GFAP and cytokeratin was 2.6% for both, adenoma and normal pituitary. However, since the total area positive for GFAP or cytokeratin was greater in pituitary tissue than in adenoma, the 2.6% of overlap equaled 44.70% of the area expressing cytokeratin and 46.77% of the GFAP positive area in adenoma, but only 15.68% vs. 24.13% of the area covered by intermediate filaments in pituitary tissue (p < 0.01 chi-square, see Table 4 in Supplement).

### Intermediate filament staining patterns

Regarding the distribution of the cells with overlapping expression of cytokeratin and GFAP, they were often found perivascular (20/26) and arranged in follicles (15/26) (Fig. 1, Table 5 in Supplement). Gonadotrophic, somatotrophic and lactotrophic adenomas showed the same pattern of predominant arrangement of co-expressing cells along vessels and in follicles (Table 7 in Supplement). In pituitary, a more diffusely scattered co-expression of cytokeratin and GFAP prevailed, although co-expressing cells were less frequently also found perivascular (2/13) and arranged as follicles (1/13) (Table 6 in Supplement).

**Fig. 2:**
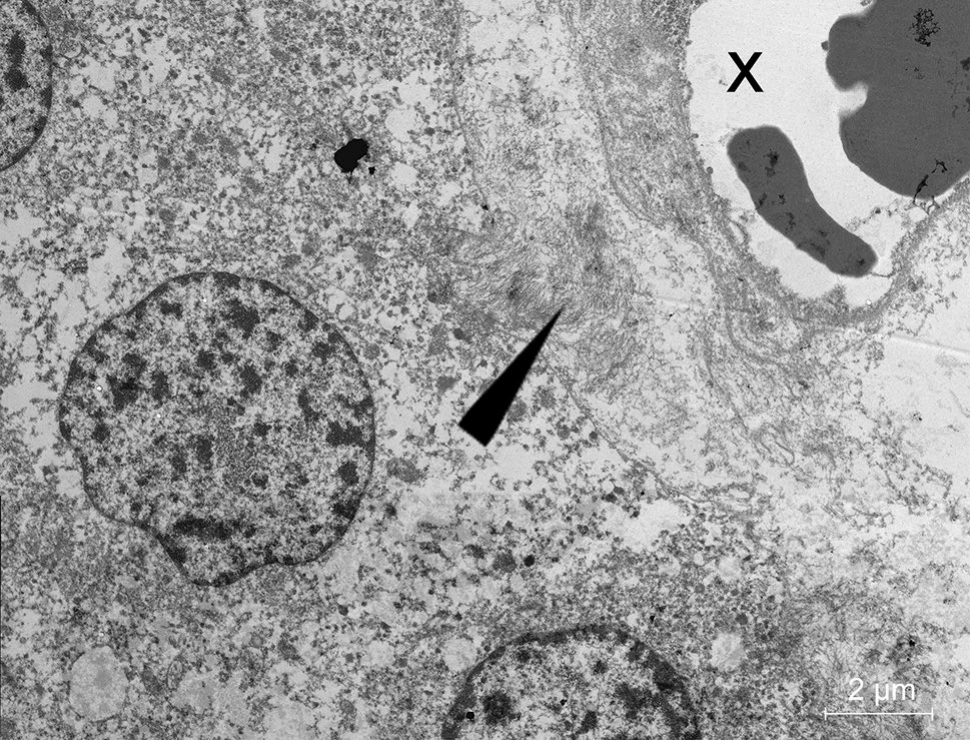
Electron microscopy depicting intermediate filaments in adenoma, arrow: intermediate filaments, X: vessel

The localization of intermediate filaments near a vessel could also be demonstrated by electron microscopy, corresponding to the findings in light and immunofluorescence microscopy (Fig. 2).

## Discussion

### Folliculostellate cells

Co-expressions of different intermediate filaments in pituitary anterior lobes and in pituitary adenomas have been described in the past [2, 7]. However, we could prove for the first time the simultaneous cellular co-expression of the intermediate filaments GFAP and cytokeratin in pituitary adenomas (Fig. 1) as well as in pituitary anterior lobes. We consider those co-expressing cells to match with folliculostellate cells described by *Rinehart and Farquhar* [3] and *Velasco *et al*.* [6]. Besides GFAP and cytokeratin, folliculostellate cells are supposed to stain for vimentin, fibronectin and S100 [12–14]. Our results are in line with the assumption that folliculostellate cells are part of the reciprocal endocrine communication system [15], as we observed that adenomas with cellular co-expression of GFAP and cytokeratin were significantly more likely to express hormones compared to adenomas without cellular co-expression of those two intermediate filaments. No null cell adenoma was found among the group of adenomas with cellular co-expression of GFAP and cytokeratin. Further, a previous study on patients with acromegaly found that higher growth hormone levels were associated with higher numbers of folliculostellate cells in the adenoma [16]. Therefore, we presume that our cells of interest play a relevant role in (paracrine) control of endocrine cells in regard of hormone synthesis and secretion [7, 10, 13, 15, 17].

Based on this assumption and correspondent with previous studies [6, 7, 18, 19], we assume that GFAP and cytokeratin co-expressing cells undergo constant morphological modification, analogous to their function and level of maturity. The morphological changes of folliculostellate cells during pathological events such as microinfarcts, mechanical interventions (surgery) or compression during growth, for instance, remind of glial reactions of the CNS. Although folliculostellate cells do not secrete hormones, they produce a number of factors and signalling molecules, such as IL-6 [13, 14, 20], vascular endothelial basic fibroblast growth factor [13, 14, 21], leukaemia inhibitory factor [14, 22], basic fibroblastic growth factor [13, 14], Annexin-1 [23], vascular endothelial growth factor [14, 24], receptors for pituitary hormones [11] and others [5]. Hence, folliculostellate cells are being stimulated by pathological events and participate at various levels of hormone production and secretion. We fortify that the GFAP and cytokeratin co-expressing cells exist in different stages, including (i) exclusive expression of GFAP, (ii) co-expression of GFAP and cytokeratin, and (iii) exclusive expression of cytokeratin (Fig. 1). It would be of interest to interrogate this hypothesis for example with the means of time-lapse recordings. As folliculostellate cells are supposed to stain for further markers (vimentin, fibronectin and S100), too, those may be included in future examinations [13].

Furthermore, after positive evidence of S-100 expression in folliculostellate cells, *Nakajima *et al*.* [8] suspected these cells to be of neuroectodermal origin. Accordingly, *Morris and Hitchcock* [7] detected the neuroectodermal marker NCAM-1 on folliculostellate cells. *Takor and Pearse* [25] suggested that due to the existence of GFAP positive cells in the epithelium of the avian Rathke cyst and their morphological similarity to the ependyma, the whole adenohypophysis is derived from the same origin as the neurohypophysis, namely from the neuroectoderm [13].

### Stem cell properties

Pituitary adenoma derived stem-like cells (PASCs) is another term for tumour stem cells in pituitary adenomas. In various studies, folliculostellate cells of the adenohypophysis have been deemed as stem cell-like [18, 22, 26–28]. *Chen *et al*.* [29] succeeded in detecting expression of stem cell antigen 1 at a high level within a side population of pituitary tissues that also contained folliculostellate cells. *Tunici and Yu* [28] uncovered nestin in cell cultures of PASCs. After incubation in a medium supplemented with growth factors (EGF and bFGF), the cell culture expressed markers of astrocytes and neurons (GFAP, β-III tubulin, and S-100).

In our studies, only 8% of all the pituitary adenomas showed evidence of GFAP and cytokeratin co-expressing cells. Since tumour stem cells may express different intermediate filaments at different stages of development, we cannot rule out that the cells we studied are PASCs, i.e. tumour-initiating cells. In regard to *Tunici*’*s and Yu*’*s* study [28], the staining of nestin on GFAP and cytokeratin co-expressing cells would thus be of interest.

### Neural transformation

As early as 1926 [30], pituitary adenomas with neural components have been described. It has been observed that endocrine neoplasias, such as pheochromocytoma [31], medullary thyroid carcinoma [32], carcinoid [33], insulin-producing pancreatic tumor [34], and small cell lung carcinoma (SCLC) [35, 36] may differentiate into neural cells in vitro. In 1986, *Martinez-Campos and Dannies* [37] induced spontaneous neural transformation of adenohypophysial cells in collagen gel. Interestingly, pituitary adenomas with neural components are most often found among the somatotrophic subtype, more rarely also among the lactotrophic and corticotrophic subtypes [19, 38]. However, as GFAP has not been detected within these neural cells of pituitary adenomas [19, 39], they seem to be not identical to the ones we observed here. In their study, *Scheithauer* et al. [38] reported prolactin-producing pituitary adenomas with positive staining for NGF receptors. Nerve growth factor (NGF) is a neurotrophic factor, known for the regulation of growth, maintenance, proliferation, and survival of neurons. Although NGF receptors can be identified in various adenohypophysial cell types, the role of NGF in the neural transformation of pituitary adenoma cells has not been well understood yet. In further studies, somatotrophic cells have been attributed adult stem cell properties, such as the ability to differentiate into diverse cell phenotypes [19]. According to *Yokoyama *et al. [40], the somatotrophic cell line preserves its progenitor cells through the influence of insulin-like growth factor 1 (IGF-1). Correspondingly, among our adenomas with GFAP cytokeratin co-expressing cells, the somatotrophic subtype represents the most frequent one. Against this background, it would be of interest to further investigate whether pituitary adenomas with GFAP cytokeratin co-expressing cells express NGF receptors and/or if they represent increasing neural elements upon the influence of IGF-1.

### Biomarker

The recurrence rate of adenomas with GFAP and cytokeratin co-expressing cells was lower than the one of pituitary adenomas without co-expression of GFAP and cytokeratin (7.7% vs. 17.8%, p < 0.05). Furthermore, there were no null cell adenomas within the cohort of GFAP cytokeratin co-expressing cells. As null cell adenomas are associated with invasive growth, adenomas with GFAP cytokeratin co-expressing cells might be less invasive. The study of *Vidal *et al*.* [19] on the neural transformation of ACTH-producing pituitary adenomas showed that adenomas with neural transformation may have a favourable prognosis. They stated a long clinical history, moderate endocrine activity related to tumour size and the absence of morphological features indicating rapid cell proliferation. The investigations of *Lange *et al*.* on the neural transformation of SCLC cells also showed that increased neuronal characteristics in vitro probably lead to a reduced malignant potential [35]. According to their results, it would be of interest whether adenomas with GFAP cytokeratin co-expressing cells are associated with a better clinical prognosis. Interestingly, they do not differ significantly from the overall cohort for Ki67 proliferation indices (1.5% vs. 1.64%).

## Electronic supplementary material

Below is the link to the electronic supplementary material.

Supplementary file1 (DOCX 21 kb)

## References

[1] Kasper M (1992). Cytokeratins in intracranial and intraspinal tissues. Adv Anat Embryol Cell Biol.

[2] Ogawa A, Sugihara S, Hasegawa M (1990). Intermediate filament expression in pituitary adenomas. Virchows Archiv B Cell Pathol.

[3] Rinehart JF, Farquhar MG (1953). Electron microscopic studies of the anterior pituitary gland. J Histochem Cytochem.

[4] Vila-Porcile E (1972). Le réseau des cellules folliculo-stellaires et des follicules de l’adénohypophyse du rat (pars distalis). Zellforsch.

[5] Fauquier T, Guérineau NC, McKinney RA, Bauer K, Mollard P (2001). Folliculostellate cells network: a route for long-distance communication in the anterior pituitary. Proc Natl Acad Sci USA.

[6] Velasco ME, Roessmann U, Gambetti P (1982). The presence of glial fibrillary acidic protein in the human pituitary gland. J Neuropathol Exp Neurol.

[7] Morris CS, Hitchcock E (1985). Immunocytochemistry in folliculo-stellate cells of normal and neoplastic human pituitary gland. J Clin Pathol.

[8] Nakajima T, Yamaguchi H, Takahashi K (1980). S100 protein in folliculostellate cells of the rat pituitary anterior lobe. Brain Res.

[9] Ishikawa H, Nogami H, Shirasawa N (1983). Novel clonal strains from adult rat anterior pituitary producing S-100 protein. Nature.

[10] Danila DC, Zhang X, Zhou Y (2000). A human pituitary tumor-derived folliculostellate cell line. J Clin Endocrinol Metab.

[11] Brokken LJ, Leendertse M, Bakker O, Wiersinga WM, Prummel MF (2004). Expression of adenohypophyseal-hormone receptors in a murine folliculo-stellate cell line. Horm Metab Res.

[12] Tsuchida T, Hruban RH, Carson BS, Phillips PC (1993). Folliculo-stellate cells in the human anterior pituitary express cytokeratin. Pathol Res Pract.

[13] Inoue K (1999). The structure and function of folliculo-stellate cells in the anterior pituitary gland. Arch Histol Cytol.

[14] Yamashita M, Qian ZR, Sano T, Horvath E, Kovacs K (2005). Immunohistochemical study on so-called follicular cells and folliculostellate cells in the human adenohypophysis. Pathol Int.

[15] Herkenham M (2005). Folliculo-stellate (FS) cells of the anterior pituitary mediate interactions between the endocrine and immune systems. Endocrinology.

[16] Voit D, Saeger W, Lüdecke DK (1999). Folliculo-stellate cells in pituitary adenomas of patients with acromegaly. Pathol Res Pract.

[17] Devnath S, Inoue K (2008). An insight to pituitary folliculo-stellate cells. J Neuroendocrinol.

[18] Horvath E, Kovacs K (2002). Folliculo-stellate cells of the human pituitary: a type of adult stem cell?. Ultrastruct Pathol.

[19] Vidal S, Horvath E, Bonert V, Shahinian HK, Kovacs K (2002). Neural transformation in a pituitary corticotroph adenoma. Acta Neuropathol.

[20] Correa-de-Santana E, Fröhlich B, Labeur M, Páez-Pereda M, Theodoropoulou M (2009). NOD2 receptors in adenopituitary folliculostellate cells: expression and function. J Endocrinol.

[21] Amano O, Yoshitake Y, Nishikawa K, Iseki S (1993). Immunocytochemical localization of basic fibroblast growth factor in the rat pituitary gland. Arch Histol Cytol.

[22] Inoue K, Mogi C, Ogawa S, Tomida M, Miyai S (2002). Are folliculo-stellate cells in the anterior pituitary gland supportive cells or organ-specific stem cells?. Arch Physiol Biochem.

[23] Theogaraj E, John CD, Christian HC, Morris JF, Smith SF (2005). Perinatal glucocorticoid treatment produces molecular, functional, and morphological changes in the anterior pituitary gland of the adult male rat. Endocrinology.

[24] Leung DW, Cachianes G, Kuang WJ, Goeddel DV, Ferrara N (1989). Vascular endothelial growth factor is a secreted angiogenic mitogen. Science.

[25] Takor T, Pearse AGE (1975). Neuroectodermal origin of avian hypothalamo-hypophyseal complex: the role of the ventral neural ridge. J Embryol Exp Morphol.

[26] Horvath E, Coire C, Kovacs K, Smyth H (2010). Folliculo-stellate cells of the human pituitary as adult stem cells: examples of their neoplastic potential. Ultrastruct Pathol.

[27] Xu Q, Yuan X, Tunici P (2009). Isolation of tumour stem-like cells from benign tumours. Br J Cancer.

[28] Tunici P, Yu JS (2009). Pituitary adenoma stem cells. Methods Mol Biol.

[29] Chen J, Hersmus N, Van Duppen V (2005). The adult pituitary contains a cell population displaying stem/progenitor cell and early embryonic characteristics. Endocrinology.

[30] Kiyono H (1926). Die Histopathologie der Hypophyse. Virchows Arch A Pathol Anat Histopathol.

[31] Franke W, Grund C, Archatatter T (1986). Co-expression of cytokines and neurofilament proteins in a permanent cell line: cultured rat PC12 cells combine neuronal and epithelial features. J Cell Biol.

[32] Tamir H, Liu K, Payette R (1989). Human medullary thyroid carcinoma: characterization of the serotonergic and neuronal properties of a neurectodermally derived cell line. J Neurosci.

[33] Ahlman H, Wigander A, Mölne J (1989). Presence of nerve growth factor-like immunoreactivity in carcinoid tumour cells and induction of a neuronal phenotype in long-term culture. Int J Cancer.

[34] Scharfmann R, Tazi A, Polak M, Kanaka C, Czernichow P (1993). Expression of functional nerve growth factor receptors in pancreatic beta-cell lines and fetal rat islets in primary culture. Diabetes.

[35] Lange A, Gustke H, Glassmeier G (2011). Neuronal differentiation by indomethacin and IBMX inhibits proliferation of small cell lung cancer cells in vitro. Lung Cancer.

[36] Zhang Z, Zhou Y, Qian H (2013). Stemness and inducing differentiation of small cell lung cancer NCI-H446 cells. Cell Death and Dis.

[37] Martinez-Campos A, Dannies PS (1986). A possible differentiation of anterior pituitary cells in collagen gels into neurons. Cell Tissue Res.

[38] Scheithauer BW, Horvath E, Kovacs K (1999). Prolactin-producing pituitary adenoma and carcinoma with neuronal components—a metaplastic lesion. Pituitary.

[39] Kontogeorgos G, Mourouti M, Kyrodimou E, Liapi-Avgeri G, Parasi E (2006). Ganglion cell containing pituitary adenomas: signs of neuronal differentiation in adenoma cells. Acta Neuropathol.

[40] Yokoyama K, Mogi C, Miura K, Kuroda K, Inoue K (2007). Somatotropes maintain their immature cells through Insulin-like growth factor I (IGF-I). Endocr Pathol.

