# Supplementary material for: Co-expression of intermediate filaments glial fibrillary acidic protein and cytokeratin in pituitary adenoma
Source: Pituitary. 2020 Oct 1;24(1):62–7. doi: 10.1007/s11102-020-01087-3 (PMC7864846; doi:10.1007/s11102-020-01087-3)
Supplement: Supplementary file 1 — Supplementary file1 (DOCX 21 kb) [file 11102_2020_1087_MOESM1_ESM.docx]

**Supplementary data “Co-expression of intermediate filaments glial fibrillary acidic protein and cytokeratin in pituitary adenoma”**

|  | **Pituitary**  **(13)** | **All adenomas**  **(326)*** | **Adenoma without co-expression of GFAP/CK**  **(264)*** | **Adenoma with**  **co-expression of GFAP/CK,**  **no co-localization**  **(36)** | **Adenoma with**  **co-expression of GFAP/CK,**  **co-localization**  **(26)** |
| --- | --- | --- | --- | --- | --- |
| **Gender** | - | M= 47.38%  (154)  F= 52.62%  (171) | M= 47.15%  (124)  F= 52.85%  (139) | M= 44.44%  (16)  F= 55.56%  (20) | M= 53.85%  (14)  F= 46.15%  (12) |
| **Mean Age** | - | 48.7 years | 49.02 years | 49.25 years | 49.3 years |
| **Recurrence rate** | - | 15.95%  (52) | 17.80%  (47) | 8.33%  (3) | 7.69%  (2) |
| **Ki 67 index** | - | 1.64% | 1.06% | 1.72% | 1.53% |

Table 1: Properties of the examined pituitary adenomas and pituitaries (control group) from 2006-2009; percentage of cases and number of cases in brackets; *: one missing value for age

|  | **Transcription**  **factor** | **All adenomas (326)** | | **No co-expression of GFAP/CK**  **(264)** | **Co-expression of GFAP/CK,**  **no co-localization**  **(36)** | | **Co-expression of GFAP/CK,**  **co-localization**  **(26)** |
| --- | --- | --- | --- | --- | --- | --- | --- |
| **Somatotrophic**  **STH, PRL** | PIT-1 | 21.78 %  (71) | 0.31 %  (1) | 18.94 %  (50) | 33.33 %  (12) | 2.78 %  (1) | 34.62 %  (9) |
| **Laktotrophic**  **PRL,(STH)** |  | 10.43 %  (34) |  | 10.61 %  (28) | 2.78 %  (1) |  | 19.23 %  (5) |
| **Thyreotrophic**  **TSH** |  | 0.31 %  (1) |  | 0.38 %  (1) | (0) |  | (0) |
| **Corticotrophic**  **ACTH** | T-PIT | 15.34 %  (50) | | 17.42 %  (46) | 8.33 %  (3) | | 3.85 %  (1) |
| **Gonadotrophic**  **FSH, TSH** | SF-1 | 24.85 %  (81) | | 25.38 %  (67) | 19.44 %  (7) | | 26.92 %  (7) |
| **Null cell adenoma** | - | 14.72%  48 | | 17.42 %  (46) | 5.56 %  (2) | | (0) |
| **Plurihormonal** | PIT-1 | 12.27 %  (40) | | 9.8 %  (26) | 27.78 %  (10) | | 15.38 %  (4) |

Table 2: Expression of the intermediate filaments GFAP and cytokeratin vs. adenoma subset; percentage of cases and number of cases in brackets

|  | **All adenomas**  **(287)** | **No co-expression of**  **GFAP/CK**  **(226)** | **Co-expression of**  **GFAP/CK,**  **no co-localization**  **(34)** | **Co-expression of**  **GFAP/CK,**  **co-localization**  **(26)** |
| --- | --- | --- | --- | --- |
| **Somatotrophic**  **STH, PRL** | 1.36 %  (66) | 1.54 %  (46) | 1.35 %  (10) | 1.2 %  (9) |
| **Laktotrophic**  **PRL,(STH)** | 3.6 %  (30) | 2.3 %  (24) | 5 %  (1) | 3.6 %  (5) |
| **Thyreotrophic**  **TSH** | 3.36 %  (1) | 3.36 %  (1) | (0) | (0) |
| **Corticotrophic**  **ACTH** | 1.62 %  (35) | 1.81 %  (31) | 2.33 %  (3) | 0.72 %  (1) |
| **Gonadotrophic**  **FSH, TSH** | 1.4 %  (79) | 1.38 %  (66) | 1.71 %  (6) | 1.1 %  (7) |
| **Null cell adenoma** | 1.46 %  (38) | 1.47 %  (34) | 1.44 %  (4) | (0) |
| **Plurihormonal** | 1.64 %  (38) | 1.77 %  (24) | 1.75 %  (10) | 1.39 %  (4) |

Table 3: Ki67 indices of the adenomas in relation to the expression of GFAP and cytokeratin as well as adenoma subset; percentage of cases and number of cases in brackets

|  | **Percentage of area with coexpression of cytokeratin and GFAP** | **Percentage of cytokeratin positive area to total area of cells with intermediate filament expression** | **Percentage of GFAP positive area to total area of cells with intermediate filament expression** |
| --- | --- | --- | --- |
| **Adenoma** | 2.6 | 44.70 | 46.77 |
| **Pituitary** | 2.6 | 15.68 | 24.13 |

Table 4: Analysis of the co-localization of GFAP and cytokeratin in the adenoma and the pituitary

|  | **Follicle** | **Perivascular** |
| --- | --- | --- |
| **Co-expression** | 15 | 20 |
| **Exclusively GFAP** | 0 | 1 |
| **Exclusively cytokeratin** | 5 | 0 |
| **Diffuse distribution of**  **co-expression** | 6 | 5 |

Table 5: Distribution patterns of co-localized GFAP and cytokeratin within the 26 adenomas samples (AE1/AE3); number of cases

|  | **Follicle** | **Perivascular** |
| --- | --- | --- |
| **Co-expression** | 1 | 2 |
| **Exclusively GFAP** | 1 | 0 |
| **Exclusively cytokeratin** | 4 | 1 |
| **Diffuse distribution of**  **co-expression** | 7 | 10 |

Table 6: Distribution patterns of co-localized GFAP and cytokeratin within the 13 pituitary samples; number of cases

|  | **Follicle** | **Perivascular** |
| --- | --- | --- |
| **Somatotrophic**  **(7)** | 0: 3  CK: 1  GFAP: 0  both: 3 | 0: 2  CK: 0  GFAP: 1  both: 4 |
| **Laktotrophic**  **(6)** | 0: 1  CK: 1  GFAP: 0  both: 4 | 0: 3  CK: 0  GFAP: 0  both: 3 |
| **Corticotrophic**  **(1)** | 0: 0  CK: 1  GFAP: 0  both: 0 | 0: 0  CK: 0  GFAP: 0  both: 1 |
| **Gonadotrophic**  **(9)** | 0: 1  CK: 1  GFAP: 0  both: 7 | 0: 0  CK: 0  GFAP: 0  both: 9 |
| **Null cell adenoma**  **(0)** | 0: 0  CK: 0  GFAP: 0  both: 0 | 0: 0  CK: 0  GFAP: 0  both: 0 |
| **Plurihormonal**  **adenoma**  **(3)** | 0: 1  CK: 1  GFAP: 0  both: 1 | 0: 0  CK: 0  GFAP: 0  both: 3 |

Table 7: Staining pattern of co-localized GFAP and cytokeratin in the 25 adenomas according to WHO classification system; 0: neither GFAP nor cytokeratin; CK: exclusively cytokeratin; GFAP: exclusively GFAP; both: cytokeratin and GFAP; number of cases
